# Fluoride and the brain: A systematic review of neurotoxicity and developmental risks

**DOI:** 10.1590/1678-4685-GMB-2025-0204

**Published:** 2026-05-15

**Authors:** Malu Siqueira Borges, Raquel Dal Sasso Freitas, Bruna Alves Alonso Martins, Fernanda Rabaioli da Silva, Ana Leticia Hilário Garcia, Juliana da Silva

**Affiliations:** 1 Universidade Federal do Rio Grande do Sul (UFRGS), Programa de Pós-Graduação em Genética e Biologia Molecular (PPGBM), Porto Alegre, RS, Brazil.; 2 Universidade La Salle (UniLaSalle), Laboratório de Genética Toxicológica, Pós-Graduação em Saúde e Desenvolvimento Humano (PPGSDH), Canoas, RS, Brazil.; 3 Cesuca Centro Universitário, Cachoeirinha, RS, Brazil.

**Keywords:** Fluoride, neurotoxicity, oxidative stress, mitochondrial dysfunction, apoptosis

## Abstract

Fluoride is a naturally occurring element found in soil, rocks, and groundwater, and its controlled use in drinking water is a well-established public health measure to prevent dental caries. However, growing evidence indicates that excessive fluoride exposure, particularly during critical periods of neurodevelopment, may exert neurotoxic effects. This systematic review evaluates current evidence on fluoride-induced neurotoxicity from *in vitro*, *in vivo*, and epidemiological studies. The findings reveal that fluoride exposure can induce oxidative stress, mitochondrial dysfunction, apoptosis, and impaired autophagy, leading to neuronal damage, synaptic deficits, and cognitive impairments. Protective mechanisms involving sirtuin proteins (SIRT1 and SIRT3) have been identified as potential modulators of fluoride-induced neurotoxicity. These results underscore the importance of monitoring fluoride exposure levels, particularly during early brain development, and lay the groundwork for future research on underlying mechanisms and preventive strategies.

## Introduction

Fluorine is the most electronegative and reactive element, a gas that does not occur in its free state in nature. In the Earth’s crust, it occurs as fluoride, being the 13th most abundant element in nature, and exists only in combination with other elements, forming fluoride compounds that are constituents of rocks and soil (Barbier et al., 2010). Its environmental concentration varies according to geological and chemical factors, such as soil composition, volcanic activity, well water depth, rock porosity, as well as anthropogenic activities (Mendoza-Schulz et al., 2009). Human exposure to fluoride occurs mainly through drinking water, food, and dental products. At optimal levels, fluoride is important in preventing dental caries and has been widely implemented as a public health measure through water fluoridation (Kimambo et al., 2019; Srivastava and Flora, 2020).

However, fluoride exposure is also associated with controversial health outcomes. While low and therapeutic levels are generally considered safe, exposure to high concentrations has been linked to adverse effects such as dental fluorosis, neurotoxicity, impaired cognitive development in children, kidney damage, and skeletal fluorosis (Zhang et al., 2007; Yang et al., 2011; Goschorska et al., 2015; Kurdi, 2016; Dec et al., 2017; Goschorska *et al*., 2018; Wei et al., 2019). Generally, these effects are associated with fluoride concentrations above 1.5 mg/L, with more severe skeletal alterations occurring at levels above 4 mg/L (Kimambo et al., 2019). The amount of fluoride permitted in drinking water, as recommended by the Word Health Organization (WHO), ranges between 0.5 and 1.5 mg/L and varies by region, taking into account other sources of exposure (WHO, 2017). Other sources of exposure include toothpaste (approximately 1000-1500 ppm) and professionally applied fluoride gel (containing an average of 5000 mg/kg) (Barbier et al., 2010). In this review, low fluoride exposure refers to concentrations at or below the WHO-recommended range (≤1.5 mg/L), therapeutic exposure refers to concentrations used in community water fluoridation programs (approximately 0.5-1.0 mg/L), and high exposure refers to concentrations exceeding these values. Exceeding these levels has raised concerns about systemic effects, including potential impacts on the central nervous system (CNS).

Evidence suggests that excessive fluoride exposure may interfere with CNS homeostasis. Experimental studies suggest that fluoride may accumulate in the hippocampus, leading to neuronal degeneration, disruption of oxygen metabolism, and overproduction of reactive oxygen species (ROS), which can trigger oxidative stress and DNA damage (Goschorska et al., 2015, 2018). These mechanisms may contribute to neuroinflammation, altered glial and neuronal metabolism, and activation of cell death pathways, impairing learning, memory, and cognitive functions (Chen et al., 2017; Duan et al., 2018; Farmus et al., 2021).

Epidemiological data from chronically exposed populations to high fluoride levels suggests a possible dose-dependent association with neurodevelopmental impairment. Studies in children from endemic areas indicate reduced intelligence quotient (IQ) and learning deficits (Chen et al., 2008; Duan et al., 2018; Lu et al., 2000). Laboratory findings further support that fluoride can cross the placental barrier, accumulate in the fetal brain, and interfere with neurodevelopment (Gui et al., 2010; Zhao et al., 2019). Gradjean’s (2019) review reinforces that elevated fluoride exposure is associated with cognitive deficits, highlighting the need for monitoring environmental concentrations in vulnerable regions. Experimental animal models also reveal structural alterations in the hippocampus and motor cortex, while *in vitro* studies indicate oxidative stress, DNA damage, and apoptosis in neural and glial cells.

Despite these findings, controversy remains. Two systematic analyses conclude that therapeutic exposure levels, such as those from community water fluoridation, are not associated with adverse neurological outcomes (Johnston and Strobel, 2020; Miranda et al., 2021). Thus, the neurotoxic potential of fluoride appears to depend on concentration, exposure window, and developmental stage of the exposed organism.

Considering the growing number of experimental and epidemiological studies, as well as the ongoing debate regarding the safety of fluoride, a systematic review is warranted. Therefore, this systematic review, conducted in accordance with the Preferred Reporting Items for Systematic Reviews and Meta-Analyses (PRISMA) guidelines and complemented by bibliometric network analysis, aims to critically evaluate the evidence linking fluoride exposure to neurotoxicity, with particular emphasis on molecular and cellular mechanisms, including oxidative stress, mitochondrial dysfunction, apoptosis, impaired autophagy, and altered protein expression in neural and glial cells.

## Material and Methods

### Selection criteria

This systematic review was conducted in accordance with PRISMA guidelines. Only peer-reviewed articles published in English were considered. Exclusion criteria comprised book chapters, books, case reports, letters to the editor, and review articles. Studies that assessed fluoride in combination with other environmental contaminants (e.g., arsenic, aluminum) or compounds with potential protective effects against fluoride toxicity were excluded. Eligible studies included those evaluating fluoride exposure alone and those investigating protein expression mechanisms using activators or inhibitors to explore fluoride-related molecular responses, including *in vitro* and *in vivo* studies.

### Search strategy

A systematic search was carried out using PubMed, Scopus, and Web of Science databases. The following search terms were applied: (fluoride OR fluorine compounds) AND (brain OR neuro*) AND human AND toxicology. The search was completed in November 2024. No time restriction applied. After duplicate removal, two independent reviewers (MSB and RDSF) screened titles and abstracts for relevance. Full texts of potentially eligible articles were then assessed for inclusion. Disagreements were resolved by consensus. All stages of screening and selection were managed using Rayyan^®^ software (Ouzzani et al., 2016).

### Co-occurrence network analysis

A bibliometric terms co-occurrence network was constructed using VOSviewer^®^ software version 1.6.20 (Center for Science and Technology Studies, Leiden University, The Netherlands) based on the studies included in this review. Terms were extracted from titles and abstracts using the full counting method. A minimum occurrence threshold of four was set to identify relevant terms. Non-specific terms were manually excluded during the refinement process.

## Results

### Results of the literature search

**Figure 1 -:**
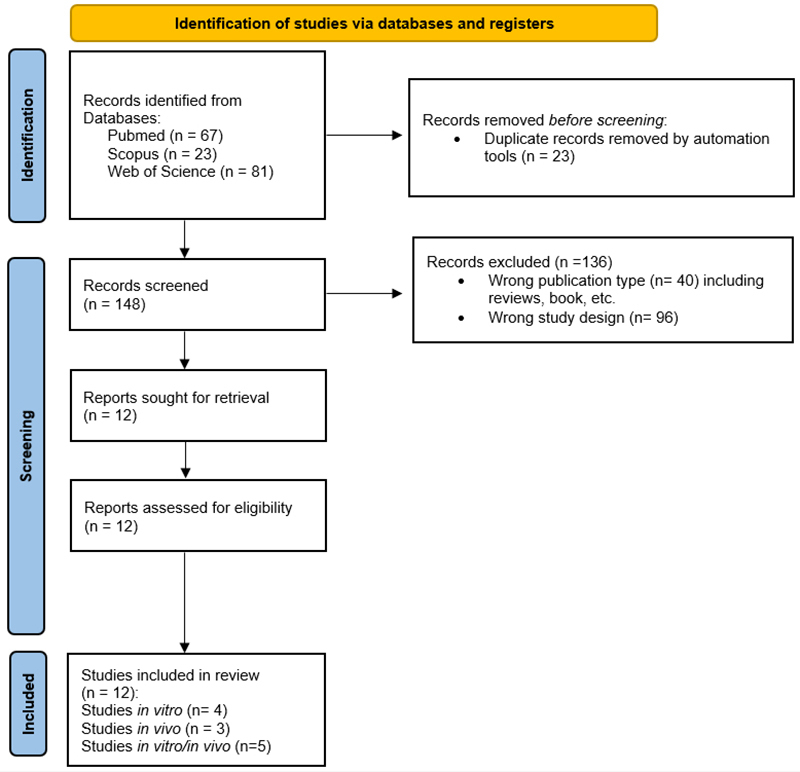
Flowchart with the results of the bibliographic search steps for the systematic review. Preferred Reporting Items for Systematic Reviews and Meta-Analyses (PRISMA) (Moher et al., 2009).

The initial search yielded 171 articles, of which 23 duplicates were removed. After screening titles and abstracts, 138 articles were excluded: 96 due to inadequate study design (e.g., studies evaluating fluoride in combination with other agents, studies that did not mention fluoride, studies with non-rat/mouse models, or studies not assessing neurotoxicity) and 40 due to publication type (case reports, review articles, letters, or books). The remaining 12 articles were selected for full review, comprising four *in vitro*, three *in vivo*, and five *in vitro/in vivo* studies (Figure 1).

### Description of the studies

*In vitro studies*

*Four studies were classified as in vitro* (Table 1). Unless otherwise specified, concentrations refer to sodium fluoride (NaF). When relevant, approximate fluoride ion (F⁻) equivalents are indicated to facilitate comparison across studies. Key findings are summarized below:

Mendoza-Schulz et al. (2009) investigated the effects of sodium fluoride (NaF) on cell migration, proliferation, and cytoskeletal dynamics in the GH4C1 rat pituitary tumor cell line. NaF concentrations ranged from 0.23 to 1072 µmol/L (corresponding approximately to 0.01-49 mg/L NaF). MTT assays showed increased metabolic activity at 2.4, 10.7, and 107 µmol/L, but a 42% reduction at the highest concentration (1072 µmol/L), indicating a biphasic, dose-dependent response. There was a reduction in the percentage of total protein content of GH4C1 in fluoride treatments. Cell migration and adhesion increased at 2.4 and 10.7 µmol/L but were impaired at 1072 µmol/L, consistent with high-dose cytotoxicity. Actin cytoskeleton analysis revealed lamellipodia formation at 1.2 µmol/L, cortical rings at 2.4 µmol/L, bleb formation at 10.7 µmol/L, and widespread actin rearrangement at 107 µmol/L, with almost no fibers at 1072 µmol/L. Western blot analysis showed variable phosphorylation of cytoskeletal proteins depending on fluoride concentration. Overall, the study demonstrated a concentration-dependent modulation of cell morphology, migration, proliferation, and cytoskeletal organization, with low doses eliciting adaptive responses and high doses inducing cytotoxic effects. 

Xu et al. (2011) examined fluoride-induced apoptosis in SH-SY5Y cells via the Fas/Fas-L pathway. Cells were treated with NaF at 0, 20, 40, and 80 mg/L (representing moderate to high exposure ranges). MTT assays demonstrated dose-dependent cytotoxicity, with significant cell death at 80 mg/L. Caspase-3 activity and mRNA levels of Fas, Fas-L, Caspase-3, and Caspase-8 increased at 40 and 80 mg/L, but not at 20 mg/L, indicating a threshold-dependent activation of apoptotic signaling. Treatment with the agonistic anti-Fas antibody CH-11 enhanced apoptosis, while the Fas-blocking antibody ZB4 partially inhibited it. Results indicate that fluoride induces apoptosis dose-dependently and that the Fas pathway is involved.

Xu et al. (2013) explored the relationship between intracellular Ca²⁺ and reactive oxygen species (ROS) during fluoride exposure in SH-SY5Y cells. Cells were treated with 20, 40, or 80 mg/L NaF (moderate to high concentrations) for 24 hours, and with 40 mg/L for various times (3-24 hours). Chelators NAC, BAPTA-AM, and EGTA were tested alone or with NaF. NaF at 40 and 80 mg/L significantly increased LDH release and ROS generation, indicating cytotoxicity Co-treatment with NAC reduced ROS and LDH while increasing Ca²⁺, EGTA increased Ca²⁺, ROS, and LDH, and BAPTA-AM reduced Ca²⁺ but altered ROS and LDH dynamics. These results suggest that fluoride-induced cytotoxicity is mediated by a complex interplay between calcium signaling and oxidative stress pathways.

Tu et al. (2018) investigated fluoride-induced apoptosis via the SIRT1/p53 pathway in SH-SY5Y cells. Cells were exposed to NaF (20-80 mg/L) (moderate to high exposure). Cell viability (CCK-8 assay) decreased significantly at ≥30 mg/L; nuclear fragmentation was observed at 40 and 60 mg/L (Hoechst staining); annexin V/PI assays confirmed increased apoptosis. Western blot analysis revealed increased cleaved caspase-3, cleaved PARP, p53, PUMA, Bax, Bcl-2, and cytochrome C in higher concentrations. Bax translocated to mitochondria and cytochrome C release into the cytoplasm were evident at 60 mg/L, indicating mitochondria-mediated apoptotic activation. Inhibition of p53 transcription reduced apoptosis, while SIRT1 overexpression or resveratrol pretreatment attenuated fluoride-induced cell death. These findings indicate that fluoride induces apoptosis via the mitochondrial p53-dependent pathway, with SIRT1 acting as a modulatory factor.

**Table 1-:** Results of *in vitro* studies involving fluoride and neurotoxicity.

Cell type	Fluoride/Fluorine/Concentrations	Significant tests and results	Reference
Pituitary tumour cells (GH4C1)	Sodium fluoride (NaF) concentrations/ (0.23; 1.2; 2.4; 10.7; 107 e 1072 µmol/L	**MTT:** ↑ 2.4; 10.7 and 107 µmol/L, and ↓ 1072 µmol/L **Western blot:** ↓ in the percentage of total protein content of GH4C1 **Cell migration and adhesion:** ↑ 2.4; 70.7 and 107 µmol/L, and 1072 µmol/L there was a loss of cell adhesion **Actin cytoskeleton arrangement:** Changes in all concentrations except the lowest **Phosphorylation of cytoskeletal proteins:** Change in phosphorylation state at different concentrations.	Mendonza-Schulz *et al*. (2009)
Human neuroblastoma cells (SH-SY5Y)	NaF concentrations (0, 20, 40, 80, 160 mg/L) other tests the maximum concentration was 80 mg/L	**MTT:** ↑ 40mg/L and 80mg/L **Percentage of apoptotic cells:** ↑ 40mg/L and 80mg/L **mRNA expression levels of Fas, Fas-L, Caspase-3, and Caspase-8:** ↑ 40mg/L and 80mg/L **0.05mg/L (CH11) + 40mg/L fluoride:** ↑ (p<0.05) in the parameters **0.5 mg/L (ZB4) + 40mg/L fluoride:** ↓ (p<0.05) in the parameters	Xu *et al*. (2011)
SH-SY5Y Cells	NaF concentrations (20, 40 and 80 mg/L - 24h), 40mg/L (3, 6, 12, 18 and 24h), and with NAC (16,32 mg/mL), BAPTA-AM (38,23 mg/mL), EGTA (380,40mg/mL) alone or with fluoride (40 mg/mL) for 12 hours	↑ LDH release in the 40 and 80 mg/L NaF groups vs control p<0.05 and p<0.05 vs 20mg/L group ↑ LDH release in the 40 mg/L NaF group at 12,18 and 24 hours vs control ↑ [Ca^2+^]i at 3, 6, 12, 18 and 24h (40 mg/L NaF) ROS ↑ at 6, 12, 18 and 24h NAC and BAPTA-AM ↓ fluoride-induced ROS and LDH levels compared to control NaF ↑ [Ca^2+^]i; ↑ ROS and LDH vs control p<0.05. NaF+NAC: ↑ [Ca^2+^]i vs control; ↓ ROS vs NaF alone; and ↓ LDH vs NaF alone. NaF+EGTA: ↑ [Ca^2+^]i vs control; ↑ ROS and LDH vs control. **NaF+BAPTA-AM**: ↓ [Ca^2+^]i vs NaF alone; ↑ ROS vs control and ↓ ROS vs NaF alone; and ↑ LDH vs control and ↓ LDH vs NaF alone	Xu *et al*. (2013)
SH-SY5Y	NaF concentrations (20; 30; 40; 50; 60; 80) other tests the maximum concentration was 60 mg/L	**CCK-8:** ↑ 40; 50; 60; 80 mg/L (viability < 50%, removed from the remaining experiments) **Morphology Hoechst:** 40 and 60 mg/L caused nuclear fragmentation and condensation. **Annexin V-FITC/PI:** ↑ apoptosis rate **Western blot:** ↑ protein expression in 40 and 60 mg/L **Immunofluorescence staining:** translocation to mitochondria after treatment with 20, 40 and 60mg/L NaF	Tu *et al*. (2018)

Legend: ↑: Increase; ↓: Decrease

*In vivo studies*

Three studies (Table 2) were classified as *in vivo*. Unless otherwise specified, concentrations refer to NaF, and when relevant, approximate F⁻ equivalents are provided. Exposure levels are described as low, therapeutic, or high according to the definitions established in the Introduction. Key findings are summarized below:

Mullenix et al. (1995) evaluated the neurotoxicity of sodium fluoride in Sprague-Dawley rats. Prenatal exposures involved injections of 0.13 mg/kg NaF, whereas post-weaning exposure was administered via drinking water containing 0-175 ppm fluoride (corresponding to a high-dose exposure range) for 6-20 weeks. Treatment with 175 ppm fluoride was discontinued after causing the death of 10 animals in 10 days, indicating severe systemic toxicity at this concentration. Adults rats received 0 or 100 ppm fluoride (high exposure) in deionized water for 5 or 6 weeks, starting at 12 weeks of age. The study also assessed behavior (stand, sit, rear, walk, and lying down) and eight modifiers (groom, head turn, look, smell, sniff, turn, wash face, and blank or no recognized activity). Three measures of spontaneous behavior were performed: calculation of behavioral initiations (BI), total behavioral time (BTT), and measurement of behavioral temporal structure (BTS), referring to the temporal distribution of the initiation of discrete acts and the sequence of joint acts. Fluoride exposure did not affect maternal weight but altered offspring behavior in a dose- and sex-dependent manner. Exposure to 100 or 125 ppm altered the behavior of males and females at 6 and 16 weeks of exposure. An increase in standing was observed in total behavioral time, and other acts decreased in initiations and total times. Brain fluoride levels increased in multiple regions with fluoride exposure through drinking water (cerebellum, hippocampus, cortex, medulla, hypothalamus, midbrain, and striatum). Exposure in adults (100 ppm for 6 weeks) increased plasma fluoride, and behavior was primarily affected in females. The study concluded that high-dose fluoride exposure is associated with region-specific brain accumulation and persistent behavioral changes.

McPherson et al. (2018) investigated the effects of prenatal and postnatal fluoride exposure in Long-Evans hooded rats. Timed-pregnant females were obtained on gestational day (GD) 4 and individually housed under controlled environmental conditions. Four groups were established, receiving diets and water with varying fluoride levels (0-20 ppm). Group 1: standard diet (20.5 ppm F-) and reverse osmosis drinking water (<0.2 ppm F-), representing a dietary fluoride background control; Group 2: low-fluoride diet (3.24 ppm F-) and reverse osmosis drinking water (<0.2 ppm F-), representing a low-exposure condition; Group 3: low-fluoride diet and water supplemented with 10 ppm F- (verified to be within 5% of the target concentration; moderate exposure); Group 4: low-fluoride diet and water supplemented with 20 ppm F- (verified to be within 5% of the target concentration; high exposure). Dosing solutions were prepared weekly using sodium fluoride (NaF). Exposure to mothers began on the 6th day of gestation and continued throughout lactation. Pups began consuming drinking water around postnatal day (PND) 14 and remained on the same exposure regimen after weaning until study termination. On the 4th postnatal day (PND), the pups from each group were transferred, cross-fostered, forming groups of 10 pups (six males and four females); the males had their nails tattooed and were randomly divided into four groups for behavioral testing (cohorts), with one male pup assigned to each endpoint, ensuring that only one male per litter was used per test. Cohort 1: exercise wheel (PND24), elevated plus maze (PND30), passive avoidance (PND55), hot plate; Cohort 2: elevated plus maze (PND29); Y-maze (PND38); Cohort 3: motor activity (PND40), light/dark place preference (PND43), Morris water maze (PND60); Cohort 4: prepulse inhibition (PND61-63), adult elevated plus maze (PND70). Behavioral assessments (running wheel, locomotor activity, plus maze, light-dark preference, hot plate latency, and Morris Water Maze) revealed minimal differences, with the most pronounced effects in the highest fluoride group (G4). Most behavioral tests showed no significant alterations, suggesting a limited behavioral impact under these exposure conditions. In adulthood (>PND90), rats exposed to 20 ppm F⁻ showed signs of mild dental fluorosis.

Sun et al. (2018) evaluated the effects of NaF exposure (0, 25, 50, 100 mg/L; representing moderate to high exposure ranges) during lactation on hippocampal glutamate receptor expression (GluR1, GluR2, NR2A, NR2B, mGluR2, mGluR5) and behavioral outcomes in mice. Behavioral tests (open field, radial arm maze) indicated that the highest concentration (100 mg/L) caused greater deficits in working memory and maze performance. NaF exposure also altered mRNA expression of hippocampal glutamate receptors, indicating disrupted synaptic signaling and neurodevelopmental interference. These findings suggest that high-dose fluoride exposure during early postnatal development impairs hippocampal-dependent cognitive functions.

**Table 2 -:** Results of *in vivo* studies involving fluoride and neurotoxicity.

***In vivo* type**	Fluoride/Fluorine/Concentrations	Significant tests and results	Reference
Sprague-Dawley rats	Injections with 0.13mg/kg of sodium fluoride (in saline solution), in pregnant rats; and 0; 75; 100; 125 or 175 ppm in drinking water, the 175 ppm concentration has been discontinued	Prenatal behavioral effect: males 17-19 days: =0.144 p<0.001 (RS statistic) **Weaning exposure:** Body weight reduction at 125 ppm exposure, both sex **Relationship between behavioral effects and plasma levels:** Females exposed for 6 weeks to 75, 100, or 125 ppm. **Behavior:** 100 or 125 ppm for males and females, and females at 6 and 16 weeks of exposure **Brain fluoride levels:** Changes in the seven regions studied with 125 ppm. Fluoride levels at 100 ppm for 6 weeks starting at 3 months of age, increased fluoride levels in the medulla oblongata in both sex and hippocampus in females	Mullenix *et al*. (1995)
Long-Evans Hooded Rats	Group 1: standard diet (20.5 ppm F-) and reverse osmosis drinking water (<0.2 ppm F-); Group 2: low-fluoride diet (3.24 ppm F-) and reverse osmosis drinking water (<0.2 ppm F-). Group 3: low-fluoride diet and water supplemented with 10 ppm F- (<5% of target); Group 4: low-fluoride diet and water supplemented with 20 ppm F- (<5% of target). Fluoride by NaF.	**Hot-plate latency at PND60:** ↓ G4 p<0.05 vs G2 **MWM; PZ and GQ:** G4 ↓ latency p< 0.05; p<0.05 and p<0.01 respectively. **Fluoride levels in rats at 25 days of age:** G1: Brain: 0.0253 (p<0.01 vs G2) and Femur: 216.7 (p<0.01). G2: Brain: 0.00 and femur: 35.2. G2 was used for comparison. G3 (10ppm): Brain: 0.0477 (p<0.01 vs G2) and femur: 235.0 (p=0.06 vs G2). G4 (20ppm): Brain: 0.0811 (p<0.01 vs G2) and femur: 379.8 (p<0.001). **Fluoride levels in adult rats:** G1: Plasma 0.018 (p<0.001 vs G2), Femur 541.6 (p<0.001 vs G2). G2: Plasma 0.001; Brain 0.21; Femur: 56.79. G3: Plasma: 0.036 (p<0.001 vs G2), Femur 681.2 (p<0.05 vs G2). G4: Plasma 0.025 (p<0.001 vs G2), Brain: 0.85 (p<0.05), Femur 993.4 (p<0.001 vs G2). TSH: G4 (18.93 p<0.01) vs G2 (2.83)	McPherson *et al*. (2018)
Kunming lineage mice	NaF (0; 25; 50 and 100 mg/L)	**increase in the total number of entries into the maze:** 100 mg/L group ↑ on days 2 to 7, 50 mg/L group ↑ on days 5 and 6. **number of working memory errors:** 100mg/L group ↑ day 2 to 6, 50mg/L group ↑ day 3, 5 and 6. **Glutamate receptor mRNA expression: GluR2:** ↓ in the 25 mg/L group (p<0.05), 50 mg/L and 100 mg/L (p<0.01); NR2A ↓ in the 50 and 100 mg/L group (p<0.05); NR2B and mGluR2 ↓ 100 mg/L group (p<0.05).	Sun *et al*. (2018)

Legend: ↑: Increase; ↓: Decrease

*In vivo/ in vitro studies*

*Five studies (* Table 3) included combined *in vivo* and *in vitro* analyses, enabling a more integrated evaluation of fluoride-induced neurotoxicity across biological systems. Unless otherwise specified, concentrations refer to NaF, and exposure levels are classified as low, therapeutic, or high based on the criteria defined in the Introduction. Key findings are summarized below:

Chen et al. (2018) assessed fluoride neurotoxicity on spinogenesis and synaptogenesis in rats and SH-SY5Y cells. Rats were treated with 0, 10, 50, or 100 mg/L NaF (representing low, moderate, and high exposure levels) via drinking water, while SH-SY5Y cells were exposed to 20-60 mg/L NaF (moderate to high exposure). Behavioral deficits were more pronounced at 50 and 100 mg/L in rats (spatial navigation test and escape latency; space probe test; swim distance). As for the *in vitro* assessments, NaF reduced SYN and PSD-95 expression, indicating impaired dendritic and synaptic formation. Pretreatment with the ERK inhibitor PD98059 rescued synaptic morphology and BDNF-TrkB signaling, highlighting the role of ERK phosphorylation as a key mediator of fluoride-induced synaptic disruption.

Zhao et al. (2019) examined mitochondrial fission/fusion imbalance in fluoride neurotoxicity in rats (gestation to 2 months postpartum), in SH-SY5Y cells, and children (8-12 years) from high- and normal-fluoride areas. Rats were treated with 0, 10, 50, or 100 mg/L NaF (low to high exposure) via drinking water), while cells were exposed to 0-60 mg/L NaF. Fluoride exposure caused cognitive deficits, mitochondrial abnormalities, defective autophagy, and increased apoptosis. Children in high-fluoride areas exhibited lower IQ scores. The study linked mitochondrial dynamics imbalance, characterized by excessive fission and impaired fusion, to fluoride-induced developmental neurotoxicity.

Zhou et al. (2019) investigated the role of autophagy in fluoride neurotoxicity and its impact on apoptosis. Sprague-Dawley rats were used as an *in vivo* model, SH-SY5Y cells were used as an *in vitro* model, and the levels of autophagy marker proteins and IQ scores of children living in areas with prolonged fluoride exposure were analyzed. Rats were divided into four groups: a control group receiving drinking water with fluoride concentrations <0.5 mg/L (low exposure), and treatment groups receiving 10, 50, or 100 mg/L NaF (moderate to high exposure). SH-SY5Y cell cultures were exposed to 0, 20, 40, and 60 mg/L NaF for 24 hours. Fluoride exposure impaired learning and memory increased cleaved caspase-3 and PARP, and suppressed autophagy (as indicated by reduced Atg5 and LC3-II levels). Furthermore, the study demonstrated that the inhibition of mTOR signaling reduced NaF-induced apoptosis and promoted cell viability in SH-SY5Y cells, suggesting that the mTOR acts as a central regulatory node in fluoride-mediated neurotoxicity. Children from endemic fluorosis areas had lower IQ, with Atg5/LC3 levels negatively correlated with fluoride levels and positively correlated with IQ.

Wang et al. (2021) explored fluoride-induced mitochondrial oxidative stress and cognitive deficits in C57BL/6 mice and SH-SY5Y cells. Mice were orally administered NaF diluted in deionized water at concentrations of 0, 25, 50, and 100 mg/L (moderate to high exposure), while SH-SY5Y cells were exposed to 110 mg/L NaF (high exposure) and genetically modified to overexpress SIRT3. NaF exposure induced significant cognitive impairments, mitochondrial dysfunction, and oxidative stress in mice, findings that were corroborated by reduced cell viability and increased oxidative markers *in vitro*. Overexpression of SIRT3 attenuated these effects, suggesting a protective role of mitochondrial antioxidant pathways in fluoride-induced neurotoxicity. 

Zhao et al. (2024) assessed SIRT1-mediated neuroprotection in SH-SY5Y cells and Sprague-Dawley rats exposed to NaF. SH-SY5Y cells were treated with 60 mg/L NaF (high exposure), while rats received drinking water supplemented with 0, 10, 50, or 100 mg/L NaF (low to high exposure). Fluoride-induced apoptosis (cleaved PARP, caspase-3) was mitigated by resveratrol and exacerbated by nicotinamide treatments, activating and inhibiting SIRT1, respectively. Mitochondrial network dynamics improved with SIRT1 activation. Bioinformatics indicated that miR-708-3p modulates SIRT1 expression, highlighting its role in neuroprotection and mitochondrial homeostasis under fluoride stress.

**Table 3 -:** Results of *in vitro/in vivo* studies involving fluoride and neurotoxicity.

***In vitro/in vivo* type**	Fluoride/Fluorine/Concentrations	Significant tests and results	Reference
Sprague-Dawley rats and SH-SY5Y cells	**Sprague-Dawley rats:** control group (drinking water with fluoride concentration lower than 1.0 mg/L), group (10 mg/L NaF), group (50 mg/L NaF) and group (100 mg/L NaF). **SH-SY5Y cells:** 20, 40, and 60 mg/L (NaF) for 24 hours.	**Sprague-Dawley: Spatial navigation test and escape latency:** ↑ in the 50 and 100mg/L groups (p<0.05) **Space probe test:** 50 mg/L NaF group: ↓ in time in the target quadrant compared to control (p<0.05) **Swim distance:** 50 and 100 mg/L groups: ↓ compared to control (p<0.05) ↓ dendritic branches and spines in exposure to 50 and 100 mg/L NaF (p<0.05) ↓ levels of the presynaptic protein SYN and the postsynaptic marker PSD-95 at 50 and 100 mg/L ↑ levels of BDNF and p-ERK1/2, while reduced TtKB at 50 and 100 mg/L NaF (p<0.05). **SH-SY5Y Cells:** ↓ SYN and PSD-95 expression levels in the 60 mg/L NaF group (p<0.05) compared to the control ↓ TrKB while ↑ BDNF in the 60 mg/L NaF group (p<0.05) vs. to the control ↑ p-ERK1/2 levels in the 40 and 60 mg/L NaF groups (p<0.05)	Chen *et al*. (2018)
Sprague-Dawley rats, SH-SY5Y cells, and Humans	**Sprague-Dawley rats:** control group (water containing less than 1.0 mg/L NaF), 10 mg/L NaF group, 50 mg/L NaF group, and 100 mg/L NaF group from gestation to 2 months postpartum. **SH-SY5Y cells:** 20, 40, and 60 mg/L (NaF) for 24 hours. **Humans:** individuals from areas with normal levels and individuals from areas with high fluoride levels	**Sprague-Dawley rats: Time of target quadrant; Distance of target quadrant; Platform crossing; Neuronal counts:** ↓ 50 and 100 mg/L NaF (p<0.05) NaF ↓ protein levels of fission regulatory molecules and ↑ levels of fusion regulators in the hippocampus of rats. It ↓ LC3 in the CA1 region in the 100 mg/L NaF group and ↑ cleaved caspase 3 in the CA1 region at 100 mg/L **SH-SY5Y cells: qRT-PCR analyses:** ↑ Drp1 in the 40 and 60 mg/L NaF group (p<0.05) ↓ Fis1 in 40 and 60 mg/L NaF (p<0.05) ↑ Mfn1 and Mfn2 in the 60 mg/L group (p<0.05) **Immunoblot:** ↓ in Fis1 and Drp1 in the 60 mg/L group (p<0.05) and ↑ in Mfn1 and Mfn2 in the 40 and 60 mg/L groups (p<0.05). **SH-SY5Y with 10 uM Mdivi-1 before treatment with 60 mg/L NaF:** ↓ in Drp1 and Fis1 and ↑ in Mfn1 and Mfn2. **NaF + Mdivi-1:** ↑ caspase 3 activation of apoptotic proteins and cleaved PARP, and ↓ cell survival. **Fis1 overexpression:** ↑fission and ↓ fusion, ↓ NaF-induced mitochondrial dysfunction, and ↓defective autophagy; ↓ apoptotic protein levels and apoptotic rate. **Humans:** Lower IQ scores in children living in areas with higher fluoride levels Circulating Fis1 levels were reduced in children in areas with higher fluoride levels Circulating Mfn2 levels were increased in children in areas with higher fluoride levels Circulating Fis1 levels were positively associated with children's IQ scores (Pearson's correlation coefficient r=0.293 p=0.035) Circulating Mfn2 levels were negatively associated with children's IQ scores (Pearson's correlation coefficient r=0.313 p=0.024)	Zhao *et al*. (2019)
Sprague-Dawley rats, SH-SY5Y cells, and Humans	**Sprague-Dawley rats:** control group (drinking water with F- concentration <0.5 mg/L); group (10 mg/L NaF); group (50 mg/L NaF); and group (100 mg/L NaF). **SH-SY5Y cells:** (20, 40, and 60 mg/L NaF) for 24 hours, to inhibit mTOR, treated with rapamycin for 1 hour. **Humans:** Cross-sectional study in Tianjin, China. 50 children (divided into 25 areas with high fluoride content and 25 areas with normal fluoride content. (Blood and urine samples were collected from the individuals)	**Escape latency and swimming distance:** ↑ in the 50 and 100 mg/L groups vs. control on days 2, 3, and 4 (p<0.01) **Spatial probe:** ↓ in the 50 and 100 mg/L groups vs. control (p<0.05) **Number of viable neurons in the CA3 region:** ↓ in the 50 and 100 mg/L groups (p<0.01) ↑ cleaved-PARP and cleaved-caspase-3 in the groups treated with 50 and 100 mg/L NaF (p<0.05) **Atg5 and LC3-II levels** ↓ in the 50 and 100 mg/L groups (p<0.05) **↑ p62 levels** in the 50 and 100 mg/L groups vs. control (p<0.05) Changes in cleaved-caspase-3 in the CA3 region of the rat hippocampus (p<0.01) Changes in Atg5 and LC3 in the CA3 region of the rat hippocampus (p<0.01) **SH-SY5Y cells:** ↓ viability in the 40 (p<0.01) and 60 mg/L NaF groups (p<0.001) **Initial and total apoptotic cell percentage** ↑ in the 40 and 60 mg/L groups (p<0.05) **Levels of cleaved-PARP and cleaved-caspase-3** ↑ in the 40 and 60 mg/L groups (p<0.05) **Levels of autophagy marker proteins:** LC3-II and Atg5 ↓ in the 40 and 60 mg/L NaF groups (p<0.05) **p62** ↑ in the 40 and 60 mg/L NaF groups (p>0.01) **Confocal microscopy:** LC3 fluorescence intensity ↓ dose-dependently (p<0.001) LC3-II levels ↓ after 2-hour exposure to 60 mg/L NaF, while changes in p-mTOR and p-p70^S6K^ levels were observed at 1 hour (all p<0.05) **LC3-II after NaF alone or combined with RAPA:** NaF+RAPA ↑ LC3-II levels and ↓ p62 levels vs. NaF alone (all p<0.05) NaF+RAPA ↓ the percentage of initial and total apoptotic cells vs. NaF alone (p<0.01) NaF+RAPA ↓cleaved PARP and cleaved caspase-3 levels vs. NaF alone (p<0.01) **CCK-8 cytotoxicity assay:** NaF+RAPA ↑ cell viability vs. NaF alone (p<0.01) **Humans:** Water F- and urine F- concentrations of children in the higher fluoride area group were ↑ vs. the control group. (p<0.001) IQ scores in the exposed group ↓ vs. the control group (p<0.001)	Zhou *et al*. (2019)
C57BL/6 mice and SH-SY5Y cells	**C57BL/6 mice:** control group; 25 mg/L NaF group; 50 mg/L NaF group and 100 mg/L NaF group. **SH-SY5Y cells:** 110 mg/L NaF; NaF+Sirt3 OE; Control (PBS)	**Time to find platform:** ↑ in the 100 mg/L NaF group vs. control **Probe test:** ↓ time in the target quadrant (100 mg/L NaF group) vs. control **Y-maze preference index:** ↓ in the 100 mg/L NaF group **Nissl staining - harmful effects on hippocampal neurons:** 100 mg/L NaF ↓ CA1 (p<0.05); CA3 ↓ (p<0.01); and Dentate gyrus (DG) ↓ (p<0.05) **Synaptic density:** ↓ 100 mg/L NaF group (p<0.05) **Enzyme activities:** SOD2 activity ↓ 100 mg/L group (p<0.001); IDH2 activity ↓ 100 mg/L NaF group (p<0.05); GSH-Px activity ↓ in the 100 mg/L NaF group (p<0.05) SOD2 acetylation level ↑ in the 50 mg/L (p<0.05) and 100 mg/L (p<0.01) groups Sirt3 expression ↓ in the 100 mg/L NaF group (p<0.01) **Expression of mtDNA-encoded genes:** ATP6: ↓ in 100 mg/L NaF group (p<0.05) vs. control; CO1: ↓ in 100 mg/L NaF group (p<0.05); Cytb: ↓ in 100 mg/L group (p<0.05); ND2: ↓ in 100 mg/L NaF group (p<0.05); ND5: ↓ in 100 mg/L NaF group (p<0.05) Western blot: FOXO3a acetylation ↑ in 50 mg/L NaF group (p<0.05) and 100 mg/L (p<0.01) Complex I activity: ↓ in 100 mg/L NaF group (p<0.05); ATP production: ↓ in 100 mg/L group (p<0.05); ROS ↑ in 50 mg/L NaF group (p<0.05) and ↑ in 100 mg/L NaF group (p<0.01); MMP ↓ in 100 mg/L NaF group (p<0.05) **SH-SY5Y cells: SOD2 activity:** ↓ 110 mg/L NaF (p<0,01) vs control, and ↑ NaF+Sirt3 OE (p<0,01) vs NaF alone; **IDH2 activity:** ↓ 110 mg/L NaF (p<0,01) vs control, and ↑ NaF+Sirt3 OE (p<0,05) vs NaF alone; **GSH-Px:** ↓ 110 mg/L NaF (p<0,01) vs control, and ↑ NaF+Sirt3 OE (p<0,05) vs NaF alone; **Acetylation of SOD2:** ↑ 110 mg/L NaF (p<0,01) vs control, and ↓ NaF+Sirt3 OE (p<0,05) vs NaF alone; **Sirt3 expression:** ↓ 110 mg/L NaF (p<0,01) vs control, and ↑ NaF+Sirt3 OE (p<0,05) **ATP6:** ↓ in 110mg/L NaF group (p<0.01) vs control; ↑ in NaF+Sirt3 EO group (p<0.05) vs NaF alone; **CO1:** ↓ in 110mg/L NaF group (p<0.01) vs control; ↑ in NaF+Sirt3 EO group (p<0.05) vs NaF alone; **Cytb:** ↓ in NaF (p<0.01) vs control; ↑ in NaF+Sirt3 EO (p<0.05) vs NaF alone; **ND5:** ↓ in NaF (p<0.01) vs control; ↑ in NaF+Sirt3 EO (p<0.05) vs NaF alone; **Western blot: Ac Fox03** ↑ in NaF (p<0.01) vs control and ↓ in NaF+Sirt3 (p<0.05) vs NaF alone; **Complex I activity:** ↓ in NaF (p<0.01) vs. control, and ↑ in NaF+Sirt3 OE (p<0.05) vs. NaF alone; **ATP production:** ↓ in NaF (p<0.01) vs. control, and ↑ in NaF+Sirt3 OE (p<0.05) vs. NaF alone; **MitoSOX Red ROS level:** ↑ in NaF group (p<0.01) vs. control, and ↓ in NaF+Sirt3 OE group (p<0.05) vs. NaF alone; **Representative dot plots of MMP (mitochondrial membrane potential):** ↓ NaF group (p<0.01) vs. control, and ↑ NaF+Sirt3 OE group (p<0.05) vs. NaF alone	Wang *et al*. (2021)
Sprague-Dawley rats and SH-SY5Y Cells	**Sprague-Dawley rats:** control group, 10mg/L group, 50 mg/L group and 100 mg/L group. **SH-SY5Y cells:** concentration of 60 mg/L	**SH-SY5Y cells:** NaF (60mg/L) + NIC: ↓ Sirt1 levels; ↓ cell survival rate; ↑ cleaved PARP and cleaved caspase-3; and cyto c overactivation ↑ by NaF+NIC vs. NaF alone **Mfn1 and Mfn2:** ↑ expression in the NaF+NIC group vs. NaF alone **Drp1 and Fis1:** ↓ expression in the NaF+NIC group vs. NaF alone **Sirt1 activation by Ad-Sirt1 and 60mg/L NaF in SH-SY5Y for 24 hours: Western immunoblot: Sirt1 protein expression levels:** ↑ in the NaF+Ad-Sirt1 group vs. NaF+vector **Mfn1 and Mfn2:** ↓ in the NaF+Ad-Sirt1 group vs. NaF+vector. **Drp1 and Fis1:** ↑ in NaF+Ad-Sirt1 vs. NaF+vector **Mitochondrial membrane potential:** ↓ NaF+vector group vs. control, and ↑ NaF+Ad-Sirt1 group vs. NaF+vector group **Apoptotic protein expression level: cyto c:** ↓ NaF+Ad-Sirt1 group vs. NaF+vector; **cleaved PARP:** ↓ NaF+Ad-Sirt1 group vs. NaF+vector **Cleaved caspase-3:** ↓ NaF+Ad-Sirt1 group vs. NaF+vector **Apoptotic rate:** ↑ NaF+vector group for early apoptotic rate vs. total apoptotic rate, and ↓ in total apoptotic rate and early apoptotic rate for the NaF+Ad-Sirt1 group vs. NaF+vector group **Survival rate:** ↓ NaF+vector group vs. control group, and ↑ NaF+Ad-Sirt1 group vs. NaF+vector group **Sprague-Dawley rats:** ↑ Sirt1 levels induced by NaF, further increased by RSV; decreased Sirt1 levels induced by NaF with NIC in the hippocampal and striatal tissues of rats compared with NaF alone **Number of crossing platforms:** ↑ NaF+RSV group vs. NaF alone group **Time spent in the target quadrant:** ↑ NaF+RSV group vs. NaF alone group **Distance spent in the target quadrant:** ↓ NaF+RSV group vs. NaF alone group **Mitochondrial area:** ↓ NaF+RSV group vs. NaF alone group, and ↑ NaF+NIC group vs. NaF alone group **Mitochondrial length:** ↑ NaF group vs. control; ↓ NaF+RSV group vs. NaF alone; and ↑ in the NaF+NIC group vs. NaF alone **Protein expression levels by Western blotting: Mfn1 and Mfn2 in the hippocampus:** ↑ NaF group vs. control; ↓ NaF+RSV group vs. NaF alone; and ↑ NaF+NIC group vs. NaF alone **Mfn1 and Mfn2 in the striatum:** ↑ NaF alone group vs. control; ↓ NaF+RSV group vs. NaF alone; ↑ in the NaF+NIC group vs. NaF alone (except for Mfn2, which was not significant) **Drp1 in the hippocampus:** ↓ NaF group vs. control; and ↑ NaF+RSV group vs. NaF alone **Fis1 in the hippocampus:** ↓ NaF group vs. control; ↑ NaF+RSV group vs. NaF alone; and ↓ NaF+NIC group vs. NaF alone **Srp1 and Fis1 striatum:** ↓ NaF alone group vs. control; ↑ NaF+RSV group vs. NaF alone group; and ↓ NaF+NIC group vs. NaF alone group **Mfn2 immunoreactivity:** ↑ NaF group vs. control; ↓ NaF+RSV group vs. NaF group; and ↑ NaF+NIC group vs. NaF alone group **Drp1 immunoreactivity:** ↓ NaF vs. control; ↑ NaF+RSV group vs. NaF alone group **Cyto chip expression level** **Hippocampus:** ↑ NaF group vs. control; ↓ NaF+RSV group vs. NaF alone; and ↑ NaF+NIC group vs. NaF alone. Striatum: ↑ NaF vs. control; ↓ NaF+RSV vs. NaF alone; and ↑ NaF+IC vs. NaF alone **Nissl staining in the CA1 region of the hippocampus:** ↓ NaF group vs. control; ↑ NaF+RSV group vs. NaF alone; and ↓ NaF+NIC group vs. NaF alone **Sit1 expression level:** miR-708-3p mimic ↓ expression in SH-SY5Y; miR-708-3p inhibitor ↓ miR-708-3p expression and ↑ Sirt1 mRNA expression, as well as ↑ relative Sirt1 protein expression levels (p<0.05) Transfection of WT Sirt1 3'UTR into HEK293 cells exhibited lower activity; luciferase ↓ in the presence of the miR-708-3p mimic vs. cells transfected with Sirt1 3'UTR ET with miR-708-3p NC. **Influence of miR-708-3p on cellular susceptibility in SH-SY5Y: Cell survival rate:** ↓ NaF group vs. the control group; ↓ NaF+miR-708-3p mimic group vs. NaF group; ↑ NaF+miR-708-3p inhibitor group vs. NaF group **SIRT1 protein expression levels:** ↑ NaF group vs. the control group; ↓ NaF+miR-708-3p mimic group vs. the NaF group **Cleaved PARP protein expression levels:** ↑ NaF group vs. the control group; ↑ NaF+miR-708-3p mimic group vs. NaF group **Sirt1 expression levels with inhibitor:** ↑ in the NaF group vs. the control group; ↑ NaF+miR-708-3p inhibitor group vs. NaF group **Expression levels of PARP and caspase-3 with inhibitor: cleaved PARP:** ↑ NaF vs. control group; ↓ NaF+miR-708-3p inhibitor group vs. NaF alone; **Cleaved caspase-3:** ↑ NaF group vs. control group and ↓ NaF+miR-708-3p inhibitor group vs. NaF group **Protein expression level of cyto C:** ↑ expression NaF group vs. control group; ↑ expression in the NaF+miR-708-3p mimic group vs. NaF alone; and ↓ expression NaF+miR-708-3p inhibitor group vs. NaF alone **Overactivation of SH-SY5Y cells with Sirt1: Western immunoblots of Sirt1:** ↓ NaF+miR-708-3p group vs. NaF alone group; ↑ NaF+miR-708-3p mimic+Ad-Sirt1 group vs. NaF+miR-708-3p mimic group **Mfn1 and Mfn2:** ↑ NaF+miR-708-3p mimic group vs. NaF alone group; ↓ NaF+miR-708-3p mimic+Ad-Sirt1 group vs. NaF+miR-708-3p mimic group **Drp1 and Fis1:** ↓ NaF+miR-708-3p mimic group vs. NaF alone group; ↑ NaF+miR-708-3p mimic+Ad-Sirt1 group vs. NaF+miR-708-3p mimic group **Cyto c and cleaved caspase-3:** ↑ NaF+miR-708-3p mimic group vs. NaF alone group; ↓ NaF+miR-708-3p mimic+Ad-Sirt1 group vs. NaF+miR-708-3p mimic group **Sirt1 expression level in SH-SY5Y cells when using inhibitor:** ↑ NaF group vs. control group; ↑ NaF+miR-708-3p inhibitor group vs. NaF alone group; ↓ NaF+miR-708-3p inhibitor+NIC group vs. NaF+miR-708-3p inhibitor group **Mfn1 and Mfn2:** ↑ NaF group vs. control group; ↓ NaF+miR-708-3p inhibitor group vs. NaF alone group; ↑ NaF+miR-708-3p inhibitor+Ad-Sirt1 group **Drp1 and Fis1:** ↓ NaF group vs. control group; ↑ NaF+miR-708-3p inhibitor group vs. NaF alone group; ↓ NaF+miR-708-3p inhibitor+NIC group vs. NaF+miR-708-3p inhibitor group **Cyto c and cleaved caspase-3:** ↑ NaF group vs. the control group; ↓ NaF+miR-708-3p inhibitor group vs. NaF alone group; ↑ NaF+miR-708-3p inhibitor+NIC group vs. NaF+miR-708-3p inhibitor group	Zhao *et al*. (2024)

Legend: ↑: Increase; ↓: Decrease

*Co-occurrence network analysis*

**Figure 2 -:**
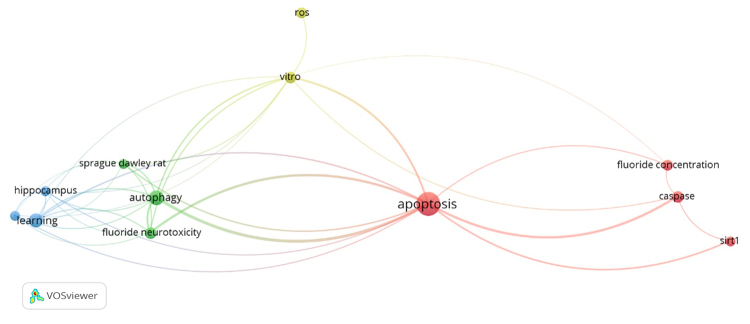
Co-occurrence network of terms extracted from the systematic review. Each term is represented by a circle whose size reflects the frequency of its occurrence throughout the 12 articles included in this systematic review. Connecting lines indicate the co-occurrence of terms within the same articles, and line thickness represents the number of articles in which the terms appear together. Clusters are distinguished by different colors, each grouping related terms.

A bibliometric co-occurrence network was based on the 12 studies included in this review. Initially, the analysis identified 440 terms, of which 34 met the minimum occurrence threshold. Among these, 20 terms (60%) were identified as the most relevant. After refinement, a final set of 12 terms was retained, distributed across four clusters and connected by 36 links. Each link represented a co-occurrence relationship, with link strength indicating the frequency of joint appearance of two terms. The total link strength metric reflected the cumulative connections of each term with all others in the network. Table 4 lists the terms found in clusters of the network formed by these 12 articles, and Figure 2 illustrates the network with the most frequently mentioned terms.

**Table 4 -:** List of selected terms extracted from the bibliometric analysis, organized by clusters.

Label	Cluster	Links	Total link strength	Occurrences
Apoptosis	1	10	214	24
Caspase		40	50	6
Fluoride concentration		3	11	5
SIRT1		2	24	4
Autophagy	2	7	124	9
Fluoride neurotoxicity		7	90	6
Sprague dawley rat		7	36	4
Hippocampus	3	7	31	4
Learning		7	49	8
Memory ability		7	32	4
ROS	4	10	70	6
Vitro		1	5	5

The most significant and frequently occurring term was “apoptosis”, indicating its central role in literature. Other relevant clusters included the green cluster, which encompassed “autophagy” and “fluoride neurotoxicity”; the blue cluster, which included “hippocampus” and “learning”; the yellow cluster, consisting of “*in vitro*” and “ROS”; and the red cluster, which contained “fluoride concentration”, “caspase”, and “SIRT1”. Strong co-occurrence relationships were observed between apoptosis and autophagy, as well as between apoptosis and caspase, highlighting the focus on cellular death mechanisms in fluoride neurotoxicity studies. Link strength analysis indicated that apoptosis had the highest total link strength, suggesting it is the most central concept across the included studies.

## Discussion

Fluoride neurotoxicity remains incompletely understood; however, the available evidence consistently suggests that fluoride can induce oxidative stress, mitochondrial dysfunction, apoptosis, and cognitive or neurodevelopmental alterations. Chronic fluoride exposure has been associated with an imbalance in ROS and antioxidant defenses, leading to oxidative stress. Xu et al. (2013) demonstrated in SH-SY5Y cells that NaF exposure increased ROS generation, LDH leakage, and intracellular Ca²⁺ release, indicating cytotoxicity. Similar findings were reported in murine models and other *in vitro* studies, where fluoride was shown to impair mitochondrial structure and function, induced swelling and cristae disorganization in hippocampal neurons, and altered the balance between mitochondrial fission and fusion (Zhao et al., 2019; Wang et al., 2021). Importantly, activation of sirtuins, particularly SIRT1 and SIRT3, attenuated fluoride-induced mitochondrial damage and oxidative stress, while inhibition of these pathways exacerbated cellular injury (Tu et al., 2018; Wang *et al*., 2021; Zhao *et al*., 2024). Altogether, these results highlight the central role of mitochondrial dysfunction and oxidative stress as early and upstream mediators of fluoride neurotoxicity.

Oxidative stress, in turn, appears to act as a major trigger of apoptotic pathways. Studies in SH-SY5Y cells consistently showed that fluoride exposure increased apoptosis in a dose-dependent manner, demonstrated by increased cleaved caspase-3 and cleaved PARP levels, increased Fas/Fas-L signaling, and mitochondrial p53 activation (Xu et al., 2011; Tu et al., 2018; Zhou et al., 2019). Moreover, overexpression or activation of SIRT1 reduced apoptosis and inhibited p53-mediated pathways, demonstrating a protective role of sirtuins against fluoride-induced neuronal death. In addition, fluoride has been shown to suppress autophagy, suggesting that disruption of cellular clearance mechanisms contributes to neuronal vulnerability (Zhou *et al*., 2019). These studies indicate that oxidative stress and mitochondrial dysfunction precede and mechanistically promote apoptotic cell death in fluoride-exposed neurons.

Cognitive and neurodevelopmental outcomes are also reflections of the neurotoxic effects of fluoride. Rodent studies have reported behavioral alterations, such as impaired learning and memory, following exposure to moderate to high fluoride concentrations, mainly during development (Mullenix et al., 1995; Chen et al., 2018; Sun et al., 2018). Fluoride exposure inhibited spinogenesis and synaptogenesis in hippocampal neurons, disrupted glutamate receptor expression, and reduced synaptic protein levels, indicating impaired neuronal connectivity (Chen *et al*., 2018; Sun *et al*., 2018). In human studies, children living in areas with elevated fluoride levels exhibited lower IQ scores than those with normal fluoride levels (Zhao et al., 2019), suggesting that the molecular and cellular effects observed *in vitro* and *in vivo* models may translate into functional deficits in humans. Nevertheless, some studies reported minimal behavioral differences at lower fluoride concentrations, supporting a concentration-dependent effect and suggesting the existence of exposure thresholds for neurodevelopmental impairment (McPherson et al., 2018).

**Figure 3 -:**
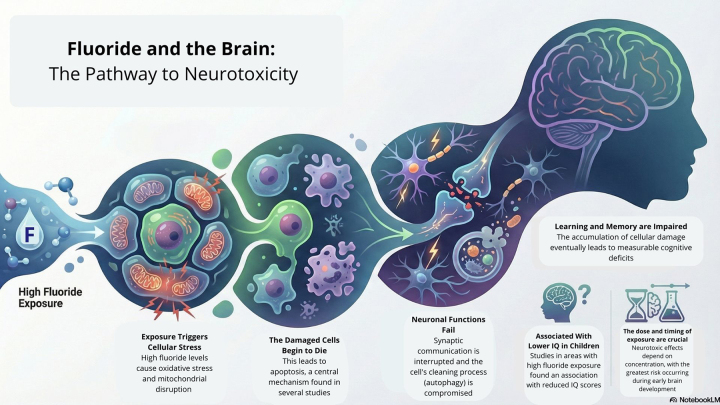
Infographic based on a systematic review. Illustrating the mechanisms by which excessive exposure to fluoride triggers a series of adverse effects on brain cells, ultimately affecting cognitive function. Image created using NotebookLM and edited with Canva.

The bibliometric analysis of the 12 studies revealed that “apoptosis” was the most frequently occurring and central term, highlighting its pivotal role in the literature on fluoride neurotoxicity. Other prominent clusters included ROS and *in vitro* models, as well as autophagy, SIRT1, and fluoride concentration, reflecting the interconnected mechanisms underlying neuronal injury. The observed co-occurrence of “apoptosis” with “autophagy” and “caspase” supports evidence that fluoride-induced oxidative stress and mitochondrial dysfunction converge on apoptotic pathways. Clusters associated with hippocampal function and learning emphasize the translational relevance of these cellular processes for cognitive and neurodevelopmental outcomes. Together, these bibliometric findings corroborate the mechanistic cascade suggested by experimental studies: fluoride exposure induces oxidative stress and mitochondrial disruption, which trigger apoptosis, impair autophagy and synaptic integrity, and ultimately compromise learning and memory (Figure 3).

The reviewed studies consistently indicate a concentration-dependent relationship between fluoride exposure and neurotoxic outcomes. *In vitro* experiments demonstrated that low to moderate NaF concentrations (1-40 mg/L or µmol/L equivalents) induced subtle changes in cell metabolism, migration, and cytoskeletal organization, whereas higher concentrations (≥60-80 mg/L) triggered apoptosis, nuclear fragmentation, caspase activation, and impaired cellular viability. Similarly, *in vivo* studies showed that higher fluoride exposures (50-100 mg/L or ppm equivalents) led to pronounced deficits in learning and memory, disrupted hippocampal synaptic structure, mitochondrial dysfunction, and increased neuronal apoptosis. In contrast, lower doses (≤20 ppm) had minimal or inconsistent behavioral effects. Furthermore, exposure during critical developmental periods amplified the neurotoxic effects of fluoride, highlighting the importance of timing in addition to dose. These concentration-dependent effects are supported by bibliometric findings, in which terms such as “fluoride concentration,” “apoptosis,” “ROS,” and “SIRT1” were central and strongly interconnected nodes, reflecting the signaling pathway in which oxidative stress and mitochondrial disruption mediate apoptosis and cognitive deficits. Collectively, the evidence underscores that both concentration and timing of fluoride exposure are key determinants of neurotoxicity, with higher concentrations and early-life exposures posing the most significant risk.

## Conclusions

This systematic review suggests that fluoride exposure induces neurotoxic effects through interconnected mechanisms, including oxidative stress, mitochondrial dysfunction, apoptosis, and impaired autophagy. These cellular and molecular alterations are frequently associated with disrupted neuronal structure, synaptic dysfunction, and deficits in cognitive performance and neurodevelopment. Bibliometric analysis further highlights apoptosis, ROS, autophagy, and SIRT1 as central concepts throughout the scientific literature, reinforcing the signaling mechanisms identified in experimental studies. The reviewed evidence also indicates a concentration- and time-dependent relationship, with higher fluoride concentrations and early-life exposures producing more pronounced cellular, molecular, and cognitive effects. The protective roles of sirtuin proteins, such as SIRT1 and SIRT3, suggest their potential as molecular modulators of fluoride-induced neuronal injury. Overall, these findings underscore the importance of considering both the dose and developmental window of exposure when evaluating the neurotoxic potential of fluoride. They also highlight the need for future studies to further elucidate the precise molecular mechanisms, exposure thresholds, and long-term consequences of fluoride exposure. From a translational perspective, these results reinforce the importance of balancing the well-established dental benefits of fluoride use with its potential neurodevelopmental risks, thereby informing evidence-based and safer public health policies.

## Data Availability

The datasets generated and/or analyzed during the current study are available from the corresponding author upon reasonable request.

## References

[B1] Barbier O, Arreola-Mendoza L, Del Razo LM (2010). Molecular mechanisms of fluoride toxicity. Chem Biol Interact.

[B2] Chen Y, Han F, Zhou Z, Zhang H, Jiao X, Zhang S, Huang M, Chang T, Dong Y (2008). Research on the intellectual development of children in high
fluoride areas. Fluoride.

[B3] Chen R, Zhao LD, Liu H, Liu HH, Ren C, Zhang P, Guo KT, Zhang HX, Geng DQ, Zhang CY (2017). Fluoride induces neuroinflammation and alters wnt signaling
pathway in BV2 microglial cells. Inflammation.

[B4] Chen J, Niu Q, Xia T, Zhou G, Li P, Zhao Q, Xu C, Dong L, Zhang S, Wang A (2018). ERK1/2-mediated disruption of BDNF-TrkB signaling causes synaptic
impairment contributing to fluoride-induced developmental
neurotoxicity. Toxicology.

[B5] Dec K, Łukomska A, Maciejewska D, Jakubczyk K, Baranowska-Bosiacka I, Chlubek D, Wąsik A, Gutowska I (2017). The influence of fluorine on the disturbances of homeostasis in
the central nervous system. Biol Trace Elem Res.

[B6] Duan Q, Jiao J, Chen X, Wang X (2018). Association between water fluoride and the level of children’s
intelligence: A dose-response meta-analysis. Public Health.

[B7] Farmus L, Till C, Green R, Hornung R, Martinez Mier EA, Ayotte P, Muckle G, Lanphear BP, Flora DB (2021). Critical windows of fluoride neurotoxicity in Canadian
children. Environ Res.

[B8] Goschorska M, Gutowska I, Olszewska M, Baranowska-Bosiacka I, Rać M, Olszowsk T, Chlubek D (2015). Effect of sodium fluoride on the catalase activity in THP-1
macrophages. Fluoride.

[B9] Goschorska M, Baranowska-Bosiacka I, Gutowska I, Metryka E, Skórka-Majewicz M, Chlubek D (2018). Potential role of fluoride in the etiopathogenesis of Alzheimer’s
Disease. Int J Mol Sci.

[B10] Gradjean P (2019). Developmental fluoride neurotoxicity: An updated
review. Environ Health.

[B11] Gui CZ, Ran LY, Li JP, Guan ZZ (2010). Changes of learning and memory ability and brain nicotinic
receptors of rat offspring with coal burning fluorosis. Neurotoxicol Teratol.

[B12] Johnston NR, Strobel SA (2020). Principles of fluoride toxicity and the cellular response: A
review. Arch Toxicol.

[B13] Kimambo V, Bhattacharya P, Mtalo F, Mtamba J, Ahmad A (2019). Fluoride occurrence in groundwater systems at global scale and
status of defluoridation - State of the art. Groundw Sustain Dev.

[B14] Kurdi MS (2016). Chronic fluorosis: The disease and its anesthetic
implications. Indian J Anaesth.

[B15] Lu Y, Sun ZR, Wu LN, Wang X, Lu W, Liu SS (2000). Effect of high-fluoride water on intelligence of
children. Fluoride.

[B16] McPherson CA, Zhang G, Gilliam R, Brar SS, Wilson R, Brix A, Picut C, Harry GJ (2018). An evaluation of neurotoxicity following fluoride exposure from
gestational through adult ages in long-Evans Hooded rats. Neurotox Res.

[B17] Mendoza-Schulz A, Solano-Agama C, Arreola-Mendoza L, Reyes-Márquez B, Barbier O, Del Razo LM, Mendoza-Garrido ME (2009). The effects of fluoride on cell migration, cell proliferation,
and cell metabolism in GH4C1 pituitary tumour cells. Toxicol Lett.

[B18] Miranda GHN, Alvarenga MOP, Ferreira MKM, Puty B, Bittencourt LO, Fagundes NCF, Pessan JP, Buzalaf MAR, Lima RR (2021). A systematic review and meta-analysis of the association between
fluoride exposure and neurological disorders. Sci Rep.

[B19] Moher D, Liberati A, Tetzlaff J, Altman DG, PRISMA Group (2009). Preferred reporting items for systematic reviews and
meta-analyses: The PRISMA statement. PLoS Med.

[B20] Mullenix PJ, Denbesten PK, Schunior A, Kernan WJ (1995). Neurotoxicity of sodium fluoride in rats. Neurotoxicol Teratol.

[B21] Ouzzani M, Hammady H, Fedorowicz Z, Elmagarmid A (2016). Rayyan-a web and mobile app for systematic
reviews. Syst Rev.

[B22] Srivastava S, Flora SJS (2020). Fluoride in drinking water and skeletal fluorosis: A review of
the global impact. Curr Environ Heal Reports.

[B23] Sun Z, Zhang Y, Xue X, Niu R, Wang J (2018). Maternal fluoride exposure during gestation and lactation
decreased learning and memory ability, and glutamate receptor mRNA
expressions of mouse pups. Hum Exp Toxicol.

[B24] Tu W, Zhang Q, Liu Y, Han L, Wang Q, Chen P, Zhang S, Wang A, Zhou X (2018). Fluoride induces apoptosis via inhibiting SIRT1 activity to
activate mitochondrial p53 pathway in human neuroblastoma SH-SY5Y
cells. Toxicol Appl Pharmacol.

[B25] Wang D, Cao L, Pan S, Wang G, Wang L, Cao N, Hao X (2021). Sirt3-mediated mitochondrial dysfunction is involved in
fluoride-induced cognitive deficits. Food Chem Toxicol.

[B26] Wei W, Pang S, Sun D (2019). The pathogenesis of endemic fluorosis: Research progress in the
last 5 years. J Cell Mol Med.

[B27] WHO - Word Health Organization (2017). Guidelines for drinking-water quality, 4th edition incorporating the
first addendum.

[B28] Xu B, Xu Z, Xia T, He P, Gao P, He W, Zhang M, Guo L, Niu Q, Wang A (2011). Effects of the Fas/Fas-L pathway on fluoride-induced apoptosis in
SH-SY5Y cells. Environ Toxicol.

[B29] Xu Z, Xu B, Xia T, He W, Gao P, Guo L, Wang Z, Niu Q, Wang A (2013). Relationship between intracellular Ca2+ and ROS during
fluoride-induced injury in SH-SY5Y cells Environ. Toxicol.

[B30] Yang S, Wang Z, Farquharson C, Alkasir R, Zahra M, Ren G, Han B (2011). Sodium fluoride induces apoptosis and alters bcl-2 family protein
expression in MC3T3-E1 osteoblastic cells. Biochem Biophys Res Commun.

[B31] Zhao Q, Niu Q, Chen J, Xia T, Zhou G, Li P, Dong L, Xu C, Tian Z, Luo C, Liu L, Zhang S, Wang A (2019). Roles of mitochondrial fission inhibition in developmental
fluoride neurotoxicity: Mechanisms of action in vitro and associations with
cognition in rats and children. Arch Toxicol.

[B32] Zhang M, Wang A, He W, He P, Xu B, Xia T, Chen X, Yang K (2007). Effects of fluoride on the expression of NCAM, oxidative stress,
and apoptosis in primary cultured hippocampal neurons. Toxicology.

[B33] Zhao Q, Zhou G, Niu Q, Chen J, Li P, Tian Z, Li D, Xia T, Zhang S, Wang A (2024). SIRT1, a target of miR-708-3p, alleviates fluoride-induced
neuronal damage via remodeling mitochondrial network
dynamics. J Adv Res.

[B34] Zhou G, Tang S, Yang L, Niu Q, Chen J, Xia T, Wang S, Wang M, Zhao Q, Liu L (2019). Effects of long-term fluoride exposure on cognitive ability and
the underlying mechanisms: Role of autophagy and its association with
apoptosis. Toxicol Appl Pharmacol.

